# The Phage Nucleus and PhuZ Spindle: Defining Features of the Subcellular Organization and Speciation of Nucleus-Forming Jumbo Phages

**DOI:** 10.3389/fmicb.2021.641317

**Published:** 2021-07-13

**Authors:** Vorrapon Chaikeeratisak, Erica A. Birkholz, Joe Pogliano

**Affiliations:** ^1^Division of Biological Sciences, University of California San Diego, La Jolla, CA, United States; ^2^Department of Biochemistry, Faculty of Science, Chulalongkorn University, Bangkok, Thailand

**Keywords:** phage nucleus, nucleus-like compartment, PhuZ, spindle-like structure, Anti CRISPR mechanism, viral speciation, subcellular organization, jumbo phage

## Abstract

Bacteriophages and their bacterial hosts are ancient organisms that have been co-evolving for billions of years. Some jumbo phages, those with a genome size larger than 200 kilobases, have recently been discovered to establish complex subcellular organization during replication. Here, we review our current understanding of jumbo phages that form a nucleus-like structure, or “Phage Nucleus,” during replication. The phage nucleus is made of a proteinaceous shell that surrounds replicating phage DNA and imparts a unique subcellular organization that is temporally and spatially controlled within bacterial host cells by a phage-encoded tubulin (PhuZ)-based spindle. This subcellular architecture serves as a replication factory for jumbo *Pseudomonas* phages and provides a selective advantage when these replicate in some host strains. Throughout the lytic cycle, the phage nucleus compartmentalizes proteins according to function and protects the phage genome from host defense mechanisms. Early during infection, the PhuZ spindle positions the newly formed phage nucleus at midcell and, later in the infection cycle, the spindle rotates the nucleus while delivering capsids and distributing them uniformly on the nuclear surface, where they dock for DNA packaging. During the co-infection of two different nucleus-forming jumbo phages in a bacterial cell, the phage nucleus establishes Subcellular Genetic Isolation that limits the potential for viral genetic exchange by physically separating co-infection genomes, and the PhuZ spindle causes Virogenesis Incompatibility, whereby interacting components from two diverging phages negatively affect phage reproduction. Thus, the phage nucleus and PhuZ spindle are defining cell biological structures that serve roles in both the life cycle of nucleus-forming jumbo phages and phage speciation.

## Introduction

Cytoskeletal structures are well known to facilitate viral replication. In eukaryotic cells, host cytoskeletal structures are utilized as a highway for viruses to travel through the cell cytoplasm in order to reach sites where they will replicate (Greber and Way, 2006; Cohen et al., 2011; Taylor et al., 2011; Portilho et al., 2016; Simpson and Yamauchi, 2020). Eukaryotic viruses also rely upon cytoskeletal proteins to travel to assembly sites before egress (Greber and Way, 2006; Taylor et al., 2011; Ward, 2011; Simpson and Yamauchi, 2020). While most eukaryotic viruses are thought to utilize the host cytoskeleton, a few are known to encode their own cytoskeletal protein. Cunha et al., 2020 recently revealed that 19 viral genomes of viruses in the family *Mimiviridae* contain actin-related genes, called viractins (Cunha et al., 2020). Viractins are conserved in the nucleocytoplasmic large DNA viruses (NCLDVs) that assemble a replication factory during its replication in the cell cytoplasm (Schmid et al., 2014; Cunha et al., 2020). Even though there is as yet no direct evidence showing the molecular interaction of viractins and nucleocytoplasmic formation, the presence of viractin sequences across the family suggests that they play a role in the NCLDV replication cycle. Some phages have also been shown to rely on bacterial host cytoskeletal proteins. *Bacillus subtilis* phage Phi29 and *Escherichia coli* phage PRD1 organize their replication machinery close to the host cell membrane by an MreB cytoskeleton-dependent mechanism (Muñoz-Espín et al., 2009, 2010).

Some phages are now known to encode homologs of the cytoskeletal protein tubulin. The best characterized of these phages are 201Phi2-1, PhiKZ, and PhiPA3. These three *Pseudomonas* phages have genomes larger than 200 kb, qualifying them as jumbo phages. Once thought to be a rarity, metagenomics studies have revealed that jumbo phages are widespread across Earth’s ecosystems, with the largest known phage genome reaching 735 kb (Al-Shayeb et al., 2020). Some of these mega phages (>500 kb in size) also encode tubulin homologs. Studies of 201Phi2-1, PhiKZ, and PhiPA3 demonstrated that the tubulin-like protein PhuZ assembles filaments that organize a replication factory contained within a nucleus-like structure (termed the phage nucleus) during infection in the bacterial host cell. Here we review the current understanding of the phage nucleus and spindle including their roles in jumbo phage replication and evolution.

## The Phage Nucleus and Phuz Spindle Establish and Maintain Complex Subcellular Organization During the Jumbo Phage Life Cycle

**FIGURE 1:**
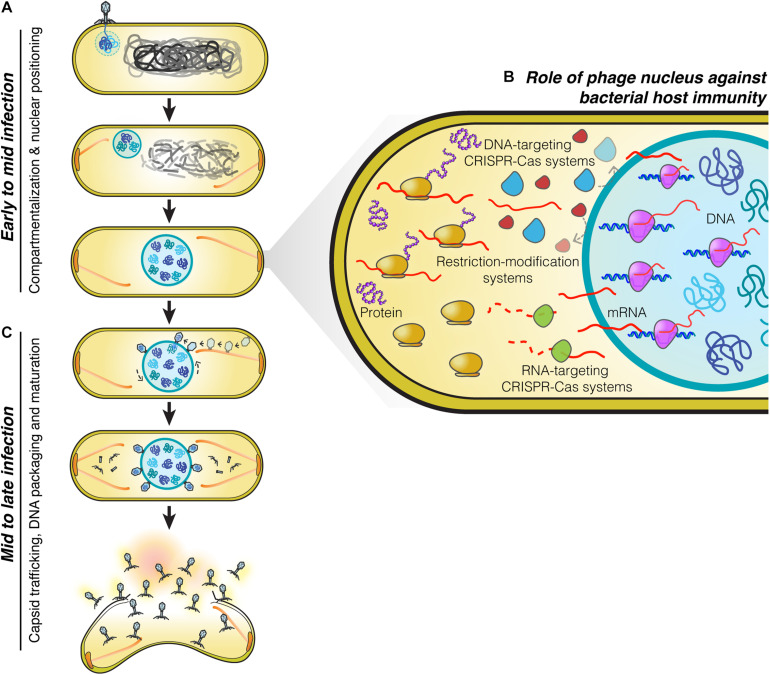
Replication machinery of nucleus-forming jumbo phages. **(A)** At the beginning of infection, phages attach to the bacterial cell membrane close to the pole and inject their genome into the cell. A nucleus-like structure is then formed by the shell protein assembling around and enclosing the phage genome. PhuZ filaments that emanate from the cell pole push the phage nucleus containing the replicating phage genome toward midcell while the host genome is degraded. When the nucleus arrives at midcell, it is met by an opposing PhuZ filament, resulting in its oscillation close to midcell. **(B)** The phage nucleus is formed by a proteinaceous shell which provides the phage genome with protection from bacterial host defense systems that target DNA because they are excluded from the shell. Phage mRNA is transported to the cell cytoplasm to initiate protein translation. Host mRNA-targeting CRISPR-Cas systems are therefore able to destroy phage mRNAs, and as a result, these phages remain sensitive to mRNA-targeting defense mechanisms (Erb et al., 2014; Chaikeeratisak et al., 2017b, 2019; Mendoza et al., 2020). **(C)** Midway through infection, procapsids assemble throughout the host cell close to the cell membrane and traffic along treadmilling PhuZ filaments toward the phage nucleus where they dock on its surface to initiate DNA packaging. Mature capsids detach from the phage nucleus and assemble with tails in the cell cytoplasm. Cell lysis occurs at the end of infection and mature phage particles are released from the cell.

The phage nucleus was originally discovered while studying phage 201Phi2-1 which replicates in *Pseudomonas chlororaphis* (Chaikeeratisak et al., 2017b), and subsequently shown to be conserved among other *Pseudomonas* phages of the PhiKZ family, including PhiPA3, and PhiKZ (Chaikeeratisak et al., 2017a). The replication cycle of PhiKZ-like viruses begins when they attach and inject their genome into the *Pseudomonas* host cell (Figure 1). Immediately after DNA injection, the phage begins expressing proteins needed for its replication. Unlike many other phages which rely upon host RNA polymerase to initiate gene expression, PhiKZ-like viruses can replicate even when the host RNA polymerases are inactivated with high concentrations of rifampicin (Ceyssens et al., 2014; Yakunina et al., 2015). These viruses encode two sets of a multi-subunit DNA-dependent RNA polymerase. One of these polymerases is thought to be packaged within the virion particle and injected into the cell along with the DNA, allowing it to initiate its program of transcriptional expression independent of the host RNA polymerase (Ceyssens et al., 2014). Like most other phages, transcription appears to be coordinately regulated into early, middle, and late gene expression, with the two different multi-subunit DNA-dependent RNA polymerase complexes likely somewhat responsible for this controlled temporal gene expression (Ceyssens et al., 2014). One of the first and most abundant proteins produced by the phage is the “shell” protein (Phi201 gp105, PhiKZ gp54, PhiPA3 gp53) that surrounds and encloses the phage genome at the site of injection (Chaikeeratisak et al., 2017a,b; Figure 1A). This encapsulated phage genome is typically located close to the cell pole, suggesting that these phages recognize a polarly localized receptor, such as the host cell flagella (Erb et al., 2014; Chaikeeratisak et al., 2017b). The PhuZ protein (Phi201 gp59, PhiKZ gp39, PhiPA3 gp28) is also expressed early and assembles dynamic filaments composed of triple stranded polymers (Kraemer et al., 2012; Erb et al., 2014; Zehr et al., 2014, 2018; Chaikeeratisak et al., 2017a,b). PhuZ filaments are polarized with plus ends that grow faster than minus ends *in vitro* (Erb et al., 2014). These filaments display both dynamic instability and treadmilling properties within the cell depending on the time point of infection (Erb et al., 2014; Chaikeeratisak et al., 2019). At the beginning of infection, when the PhuZ concentration is relatively low, these dynamic filaments assemble spindle-like structures at cell poles by an unknown mechanism. The minus ends are thought to be anchored to the cell pole leaving the plus ends pointing toward the cell center. As the filaments grow and shrink over time, they push the expanding phage nucleus toward midcell. When it reaches the cell midpoint, the filaments from the opposite site push it back, resulting in the oscillation of the nucleus close to midcell (Erb et al., 2014; Chaikeeratisak et al., 2017b; Figure 1A). Soon after infection, the host chromosome is degraded (Erb et al., 2014), potentially to provide free nucleotides for new phage DNA, as well as space for the phage nucleus.

On the journey to midcell, the phage nucleus continues to enlarge in size as the phage genome replicates (Kraemer et al., 2012; Erb et al., 2014; Chaikeeratisak et al., 2017b). Surprisingly, the shell of the nucleus has a selective property and thus it can compartmentalize proteins according to function (Chaikeeratisak et al., 2017b). In general, proteins needed for phage DNA replication, transcription, recombination, or repair are found to specifically localize inside the phage nucleus along with the phage DNA. In contrast, metabolic enzymes, such as thymidine kinase produced by the phage, and ribosomes of the host cell are excluded from the phage nucleus (Chaikeeratisak et al., 2017b). Host DNA topoisomerase, which serves a role in DNA replication, is hijacked by the phage as it is localized inside the phage nucleus (Chaikeeratisak et al., 2017b). The mechanisms underlying selective protein transport that allow specific proteins to be targeted inside the nucleus while other proteins are excluded currently remain unknown.

The phage nucleus has the ability to perform the same key functions as the eukaryotic nucleus, namely, separating DNA from the cytoplasm, which partitions DNA replication and transcription away from translation and metabolic enzymes. Another similarity to the eukaryotic nucleus is that phage mRNAs are produced within the nucleus and must be transported to the cytoplasm where the ribosomes are located, in order to initiate protein synthesis (Chaikeeratisak et al., 2017b; Figure 1B). Proteins involved in DNA replication, recombination, and transcription are then selectively imported into the nucleus. Although the mechanisms have yet to be elucidated, the shell must contain pores that allow proteins to selectively enter and also allow for mRNA molecules to exit and reach the cytoplasm. As in eukaryotes, this two-way exchange of macromolecules must be essential for allowing the establishment of this extraordinarily complex subcellular organization. While the molecular architecture of the barriers that achieve DNA compartmentalization for the eukaryotic nucleus and the phage nucleus are different, with one composed of a double membrane scaffolded with a proteinaceous layer called the lamina, and the other of a single layer of protein, they both achieve the same subcellular organization with the uncoupling of transcription from translation, mRNA export, and selective protein import.

## Mid to Late Infection: Capsid Trafficking, DNA Packaging and Maturation

Phage maturation of PhiKZ-like viruses starts to occur after approximately 40 minutes post-infection (mpi) (Chaikeeratisak et al., 2017b, 2019). At this stage, the bipolar spindles have already positioned the nucleus at midcell (Kraemer et al., 2012; Chaikeeratisak et al., 2017b, 2019; Figure 1C). Phage procapsids begin assembling at approximately 45 mpi (Chaikeeratisak et al., 2017b, 2019). These procapsids assemble randomly throughout the bacterial cell membrane and later attach to the spindles (Chaikeeratisak et al., 2019), which begin to exhibit treadmilling activity (Chaikeeratisak et al., 2019). The fully assembled procapsids then traffic along the treadmilling filaments toward the nucleus and, when they reach the nuclear shell, they dock onto the surface for DNA encapsidation (Chaikeeratisak et al., 2019). Our cryo-FIB-ET tomograms revealed a small gap (∼ 3–4 nm) at the site between the procapsids and the filaments (Chaikeeratisak et al., 2019), suggesting the possible presence of an adaptor protein that is involved in the transport of procapsids (Chaikeeratisak et al., 2019).

When procapsids reach the phage nucleus at the depolymerizing plus end of the filament, they dock onto the nuclear surface and initiate the DNA packaging process. As the incoming procapsid arrives at the same subcellular location as the previous procapsid, the PhuZ spindle also rotates the phage nucleus when the two filament ends of the spindle from each side push the structure transversely in order to provide a new surface for docking (Chaikeeratisak et al., 2019; Figure 1C). This rotation mechanism driven by the spindle is necessary to distribute the procapsids around the nucleus which will maximize the efficiency of phage DNA packaging. The processes of capsid transport and nuclear rotation are important for the maximum rate of phage reproduction. In the presence of catalytic-defective PhuZ filaments that are unable to exhibit treadmilling activity, procapsids are not transported to the nucleus but instead are trapped along the static filaments (Chaikeeratisak et al., 2019), vastly diminishing the rate of successful DNA packaging. Whether PhuZ spindles serve a role in spatial organization of additional steps of mature phage assembly is unknown and requires further investigation.

## The Phage Nucleus and Tubulin Spindle Are Conserved Among Jumbo Phages

The phage nucleus and PhuZ spindle are conserved among jumbo phages based on the experimental confirmation of these structures in *Pseudomonas* PhiKZ-like viruses: 201Phi2-1, PhiPA3, and PhiKZ (Chaikeeratisak et al., 2017a). Bioinformatic analysis showed that the genes encoding the shell and PhuZ proteins are also found in *Serratia*, *Erwinia*, *Ralstonia*, and *Vibrio* phages that have a genome larger than 200 kb (Chaikeeratisak et al., 2017a, 2019; Malone et al., 2020). *Serratia* phage PCH45 has been shown to assemble a centrally positioned nuclear-like structure during lytic replication (Malone et al., 2020). Homologs of the shell and PhuZ proteins are also found in uncultured large phages identified in metagenomic studies (Al-Shayeb et al., 2020). These findings suggest the widespread importance of subcellular organization during reproduction of large phages.

## Role of the Phage Nucleus Against Bacterial Host Immunity

Phages and bacteria have been co-evolving for more than a billion years (Brüssow et al., 2004). As a defense mechanism against phages, bacteria have evolved a large number of strategies to counter phage infection, including restriction modification systems and an adaptive immune system known as clustered regularly interspaced short palindromic repeats (CRISPRs) and their associated Cas enzymes that can specifically target and destroy phage genetic material in order to prevent the viral infection (Barrangou et al., 2007; Labrie et al., 2010; Jiang and Doudna, 2017; Nussenzweig and Marraffini, 2020). The ability of the phage nucleus to exclude host enzymes suggested that it provides protection from host DNA targeting enzymes (Chaikeeratisak et al., 2017b). This was recently confirmed for phage PhiKZ, whose nucleus excludes multiple types of CRISPR-associated enzymes (cas3, cas9, and cas12a) and the type I restriction enzymes on the outside (Mendoza et al., 2020; Figure 1B). Genome protection by the phage nucleus has also been observed in a distantly related phage, phage PCH45, which infects and replicates in *Serratia*. The PCH45 phage nucleus broadly protects its genome by excluding three different native CRISPR-Cas complexes in *Serratia* (Malone et al., 2020). Since phage mRNA must be transported to the cell cytoplasm to reach the ribosomes for translation (Chaikeeratisak et al., 2017b), these phages are still susceptible to RNA-targeting CRISPR-Cas systems resulting in infection arrest (Malone et al., 2020; Mendoza et al., 2020; Figure 1B). Given the presence of shell homologs in many other jumbo phages (Chaikeeratisak et al., 2017a; Malone et al., 2020; Mendoza et al., 2020), the phage nucleus as a mechanism to overcome bacterial host immunity is likely widespread.

## Role of Subcellular Genetic Isolation and Virogenesis Incompatibility in Viral Speciation

**FIGURE 2:**
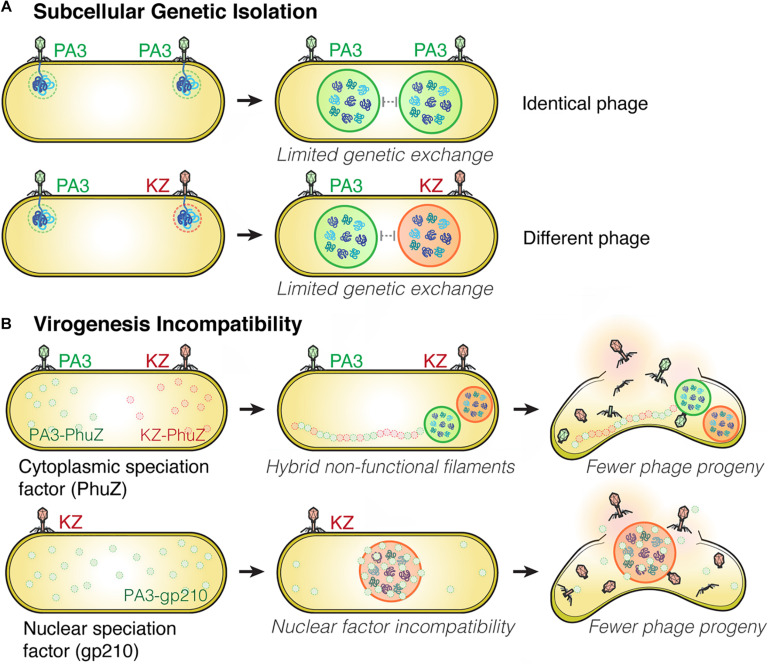
Subcellular Genetic Isolation and Virogenesis Incompatibility contribute to viral speciation. **(A)** During co-infection by either identical (such as two PhiPA3) or different phages (PhiPA3 and PhiKZ), each individual phage assembles its own compartment that physically separates its genome from another, resulting in Subcellular Genetic Isolation. In both cases of co-infection, with phages that are either identical or different, the shell potentially limits genetic exchange promoting evolutionary divergence. **(B)** Virogenesis Incompatibility occurs when divergent components of phage replication interact negatively, causing interference with viral replication, such as PhuZ (a cytoplasmic speciation factor) and gp210 (a nuclear speciation factor). This incompatibility results in a reduction in phage fitness (Chaikeeratisak et al., 2021).

Viruses are known to have very high rates of genetic exchange that can occur when two phages infect the same host cell (Bobay and Ochman, 2018; Chaikeeratisak et al., 2021). Recent studies suggest co-infections of bacteria are very common in natural ecosystems (Díaz-Muñoz, 2017; Luque and Silveira, 2020). Host specificity is therefore known to be one of the key factors that limits genetic exchange among two phages and allows two strains to diverge from one another (Duffy et al., 2007; Meyer et al., 2016; Saxenhofer et al., 2019). We recently studied nucleus-forming jumbo phages and discovered several speciation factors belonging to two general mechanisms of viral speciation (Chaikeeratisak et al., 2021; Figure 2). First, we found that the proteinaceous shell which guards phage genetic material from host immunity also forms a barrier that reduces the likelihood of genetic exchange between phages during co-infection (Figure 2A). When a single *P. aeruginosa* cell was simultaneously infected with jumbo phage PhiKZ and PhiPA3, two distinct nuclei physically separating the two phage genomes from each other were formed in the majority of co-infections, thereby potentially limiting genetic exchange between them. This subcellular isolation also occurred when the cell was co-infected with a single species of phage (either PhiKZ or PhiPA3). The phage nucleus thus establishes “Subcellular Genetic Isolation,” a condition limiting the opportunity for co-infecting viruses to recombine due to subcellular spatial restrictions (Chaikeeratisak et al., 2021; Figure 2A). Subcellular Genetic Isolation is likely a widespread mechanism that limits genetic exchange among viruses and therefore contributes to their species diversity (Schmid et al., 2014). For example, other viruses, including herpesvirus (Tomer et al., 2019), poxvirus (Kieser et al., 2020) and nucleocytoplasmic large DNA viruses (NCLDVs) (Mutsafi et al., 2014), assemble physically separated replication factories during replication. Herpesviruses were shown to undergo limited recombination when replication factories were isolated from each other but increased recombination when they coalesced (Tomer et al., 2019). Thus, for viruses that replicate in localized factories, even within cells as small as a bacterium, Subcellular Genetic Isolation might come into play as a mechanism that allows for evolutionary divergence.

Another mechanism that limits the potential for genetic exchange between jumbo phages is “Virogenesis Incompatibility,” in which components from two distinct phages interact to negatively affect phage reproduction (Chaikeeratisak et al., 2021; Figure 2B). We have identified two Virogenesis Incompatibility factors for the nucleus-forming jumbo phages. The phage spindle, which is responsible for the spatial and temporal subcellular organization during phage maturation, becomes a Virogenesis Incompatibility factor when the PhuZ proteins from two different phages diverge and become incompatible with each other. For example, PhuZ proteins of phage PhiKZ and PhiPA3 share limited sequence identity (37%), yet they co-assemble to form a hybrid filament during co-infection. However, the hybrid PhiKZ-PhiPA3 filament has lost all dynamic properties, and thus is non-functional (Chaikeeratisak et al., 2021; Figure 2B). Since the PhuZ spindle plays many crucial roles in phage development such as nuclear positioning (Kraemer et al., 2012; Erb et al., 2014; Chaikeeratisak et al., 2017b), nuclear rotation (Chaikeeratisak et al., 2017b, 2019), and capsid trafficking (Chaikeeratisak et al., 2019), the hybrid filaments interfere with the replication of both infecting species, which results in smaller and mispositioned nuclei, and a ∼ 50% decrease in the number of phage offspring (Chaikeeratisak et al., 2019, 2021).

A second Virogenesis Incompatibility factor is the PhiPA3 protein gp210, a nuclease which normally resides within the PhiPA3 nucleus. Transporting PhiPA3 gp210 into the PhiKZ nucleus greatly reduces PhiKZ production, indicating the incompatibility of this protein with the production of PhiKZ particles. PhiPA3 gp210 is a Virogenesis Incompatibility factor that could contribute to the divergence of PhiPA3 and PhiKZ by specifically reducing PhiKZ production under conditions in which PhiPA3 and PhiKZ formed a single, shared nucleus during co-infection (Chaikeeratisak et al., 2021; Figure 2B). Thus, the phage nucleus plays a key role in viral speciation by providing Subcellular Genetic Isolation that directly limits genetic exchange and by providing protection from viral nuclear incompatibility factors that would otherwise result in Virogenesis Incompatibility.

Similar to Subcellular Genetic Isolation, we expect Virogenesis Incompatibility to also be widespread among both eukaryotic and prokaryotic viruses. Since presumably all viruses require the production of virion particles from a limited set of self-assembling components, such as capsids, tails, tail fibers, portals, terminases, nuclear shells, PhuZ spindles, etc., divergence between any of these polymerizing proteins can potentially result in Virogenesis Incompatibility during co-infection, as we have demonstrated for the PhuZ spindle. Virogenesis Incompatibility has also been reported for eukaryotic segmented viruses: influenza A and B (Frank, 2001; Baker et al., 2014; White and Lowen, 2018). Consisting of 8 individual segments, the influenza A genome is shuffled during replication. When two viruses co-infect the same cell, shuffling can result in genetic incompatibilities when a capsid packages incompatible genome segments from different parental viruses (such as H5N8 and H9N2) (Mostafa et al., 2020), resulting in the production of fewer functional progeny. Although the underlying mechanisms are not yet understood, this evidence suggests that Virogenesis Incompatibility is also widespread.

## Concluding Remarks

The eukaryotic nucleus is the defining structure of eukaryotic cells. It is a membrane-bound organelle with a lamina protein scaffold inside that separates genomic DNA from the cytoplasm and serves the key purpose of uncoupling transcription and translation (Lammerding, 2011). Recently, various subcellular structures analogous to the eukaryotic cell nucleus have been discovered. For example, Planctomycetes appear to possess a “bacterial nucleus” (Fuerst, 2005; Hendrickson and Poole, 2018) that is formed by irregularly shaped cytoplasmic membranes that partially surround the cell nucleoids (Boedeker et al., 2017; Wiegand et al., 2018). This structure was originally postulated to uncouple transcription machinery from translation of mRNA in the cell cytoplasm (Fuerst, 2005). More recent work suggests that ribosomes are found within the Planctomycetes nucleus-like structure (Fuerst, 2005; Hendrickson and Poole, 2018; Jogler et al., 2019). It has been proposed that there is a larger proportion of active ribosomes distributed distantly from the nucleoid than adjacent to it (Gottshall et al., 2014). Due to the existence of ribosomes inside the Planctomycete nucleus-like compartment, whether or not this compartment in Planctomycetes is truly nucleus-like is still an ongoing debate (Gottshall et al., 2014; Hendrickson and Poole, 2018; Jogler et al., 2019; Poole and Hendrickson, 2019). A replication compartment has also been reported in eukaryotic viruses. During infection, the NCLDV viruses recruit internal membrane from host endoplasmic reticulum to establish their own subcellular compartment to serve as a viral replication factory in the host cytoplasm (Schmid et al., 2014; Hendrickson and Poole, 2018). This viral nucleus-like structure, particularly from Mimivirus, contains DNA replication and RNA transcription-related machinery and excludes ribosomes (Fridmann-Sirkis et al., 2016). NCLDV viruses have also evolved complex pathways involved in mRNA processing and nuclear export as in eukaryotic cellular systems, supporting the Viral Eukaryogenesis Theory (Schmid et al., 2014; Bell, 2020). In comparison, the nucleus-like structure formed by jumbo phages efficiently separates transcription and translation using a proteinaceous shell instead of a lipid bilayer (Chaikeeratisak et al., 2017a,b). While the phage nucleus is structurally unrelated to the eukaryotic nucleus, remarkably, it is able to achieve the similar function of separating transcription from translation while allowing the two-way exchange of proteins and metabolites. Nucleus-forming jumbo phages also harbor a PhuZ cytoskeleton that is evolutionarily related to the eukaryotic tubulin cytoskeleton and that has dynamic properties similar to the eukaryotic spindle (Kraemer et al., 2012; Erb et al., 2014). These subcellular structures are conserved among the nucleus-forming jumbo phages and support the Viral Eukaryogenesis Theory by showing that viruses can evolve similar structures (Chaikeeratisak et al., 2017a; Bell, 2020). The functions of the phage nucleus together with the PhuZ spindle might provide insight into how these types of structures might evolve.

## Author Contributions

VC, EB, and JP contributed on the conception and flow of this minireview, wrote the draft, revised the manuscript, and approved it for publication. All authors contributed to the article and approved the submitted version.

## Conflict of Interest

The authors declare that the research was conducted in the absence of any commercial or financial relationships that could be construed as a potential conflict of interest.
